# Is repeat serum urate testing superior to a single test to predict incident gout over time?

**DOI:** 10.1371/journal.pone.0263175

**Published:** 2022-02-01

**Authors:** Sarah Stewart, Amanda Phipps-Green, Greg D. Gamble, Lisa K. Stamp, William J. Taylor, Tuhina Neogi, Tony R. Merriman, Nicola Dalbeth

**Affiliations:** 1 Department of Medicine, University of Auckland, Grafton, Auckland, New Zealand; 2 Department of Medicine, University of Otago Dunedin, Dunedin Central, Dunedin, New Zealand; 3 Department of Medicine, University of Otago Christchurch, Christchurch Central City, Christchurch, New Zealand; 4 Department of Medicine, University of Otago Wellington, Newtown, Wellington, New Zealand; 5 School of Medicine, Boston University Medical School, Boston, Massachusetts, United States of America; Holbaek Sygehus, DENMARK

## Abstract

Elevated serum urate is the most important causal risk factor for developing gout. However, in longitudinal cohort studies, a small proportion of people with normal urate levels develop gout and the majority of those with high urate levels do not. These observations may be due to subsequent variations in serum urate over time. Our analysis examined whether single or repeat testing of serum urate more accurately predicts incident gout over time. Individual participant data from three publicly-available cohorts were included. Data from paired serum urate measures 3–5 years apart, followed by an assessment of gout incidence 5–6 years from the second urate measure were used to calculate the predictive ability of four measures of serum urate on incident gout: the first measure, the second measure, the average of the two measures, and the highest of the two measures. Participants with prevalent gout prior to the second measure were excluded. Receiver operator characteristic (ROC) curves and area under the curve (AUC) statistics were computed to compare the four measures. A total of 16,017 participants were included across the three cohorts, with a mean follow-up from the first serum urate test of 9.3 years (range 8.9–10.1 years). Overall, there was a small increase in the mean serum urate between the first and second measures (322 μmol/L (5.42 mg/dL) vs. 340 μmol/L (5.71 mg/dL), P<0.001) which were a mean of 3.5 years apart, but the first and second measures were highly correlated (r = 0.81, P<0.001). No differences were observed in the predictive ability of incident gout between the four measures of serum urate measurement with ROC curve AUC statistics ranging between 0.81 (95% confidence intervals: 0.78–0.84) and 0.84 (95% confidence intervals: 0.81–0.87). These data show that repeat serum urate testing is not superior to a single measure of serum urate for prediction of incident gout over approximately one decade.

## Introduction

Elevated serum urate concentration (hyperuricemia) is the most important risk factor for developing gout [1–3], with a strong concentration-dependent relationship between serum urate levels and incidence of gout [1–5]. However, in longitudinal cohort studies, baseline serum urate does not fully predict development of gout; a small proportion of people with normal urate levels develop gout, and the majority of those with hyperuricemia at baseline do not [1–3, 5]. These observations may be due to subsequent variations in serum urate over time.

Serum urate levels can vary within individuals over time [2, 6], and practitioners may monitor this variation through repeat testing, in order to improve the ability to accurately predict development of gout, particularly in individuals who are at a higher risk for hyperuricemia and gout (i.e., family history). However, as with any laboratory-based serum test, repeat testing of serum urate places additional burden on the individual, including work absences to attend appointments, as well as a financial burden related to health-care costs. The aim of this analysis was to examine whether single or repeat testing of serum urate more accurately predicts incident gout over time.

## Methods

### Cohorts

Three US cohorts with publicly-available data were used in this analysis; Atherosclerosis Risk in Communities Study (ARIC) [7], Coronary Artery Risk Development in Young Adults Study (CARDIA) [8], and the original cohort of the Framingham Heart Study (FHS) [9] (S1 Fig). Data from the offspring cohort of the FHS study were excluded from this analysis as they did not meet the below criteria for inclusion (lacked paired urate assessment three to five years apart that were followed by an assessment of gout development at a study visit within five to six years of the second urate measurement). Database of Genotype and Phenotype approval number was 834.

**Fig 1:**
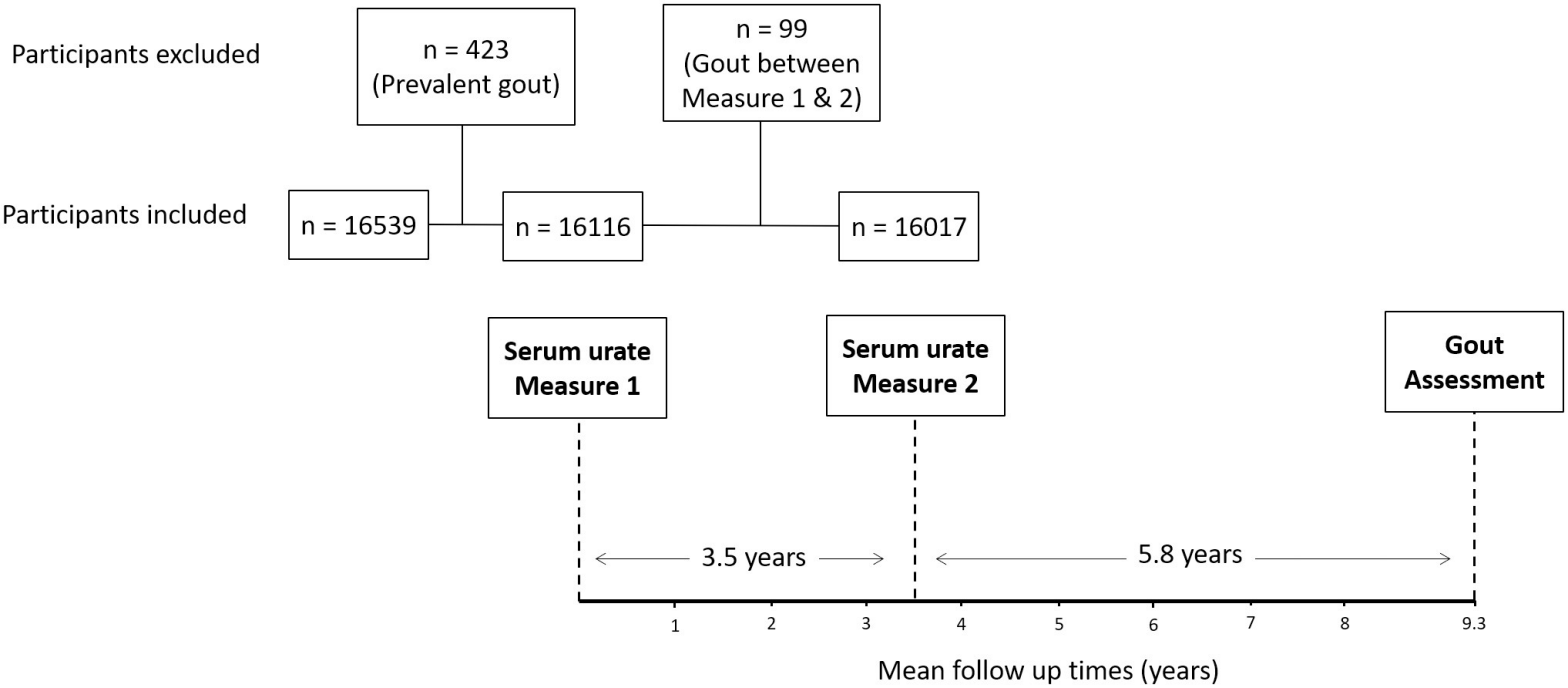
Study timeline, and flow of study participants in the analysis.

Paired urate assessments (three to five years apart) included in the analysis were selected if they were followed by an assessment of gout development at a study visit within five to six years of the second urate measure. Data from ARIC were included from 1987 to 1989 (urate Measure 1), 1990 to 1992 (urate Measure 2) and 1996 to 1998 (Gout Assessment). Data from CARDIA were included from 1995 to 1996 (Measure 1), 2000 to 2001 (Measure 2) and 2005 to 2006 (Gout Assessment). From FHS, data were included from 1950 to 1955 (Measure 1), 1954 to 1958 (Measure 2) and 1960 to 1964 (Gout Assessment). ARIC and CARDIA used a standard uricase oxidation assay to measure serum urate, while FHS study used a phosphotungstic acid reagent autoanalyzer to measure serum urate. Only data from participants who were free from gout before the two time points of serum urate measurement (Measure 1 and Measure 2) were included. For all three cohorts, gout status was determined by a self-reported diagnosis of gout ascertained at the study visits. Self-reported diagnosis of gout has been validated in and analysis of definitions of gout for use in epidemiological studies; in this analysis, self-report had a similar performance to the widely-used 1977 ARA gout classification criteria, with high sensitivity (80%) and specificity (72%) compared to gold standard monosodium urate crystal identification [10]. Details of the three cohorts and clinical and demographic characteristics of the participants included (n = 16,017) and excluded (n = 522) in the current analysis are shown in S1 Table. Excluded participants consisted of 423 who developed gout prior to or at Measure 1, and 99 who developed gout between Measure 1 and Measure 2 (Fig 1). Mean (SD) serum urate at Measure 1 was higher in excluded participants compared to included participants (7.4 (1.9) vs. 5.4 (1.5), respectively) (S1 Table).

### Data analysis

Initially, to determine whether there was a difference in mean serum urate between Measure 1 and Measure 2, a mixed linear regression model was used with serum urate as the outcome variable and Measure (1 or 2) as a predictor variable. The cohort (ARIC/CARDIA/FHS) was also included as a factor in order to account for any heterogeneity across the cohorts. Pearson’s r correlation coefficients were used to compute the correlation between serum urate at Measure 1 and Measure 2 To assess potential regression to the mean in serum urate measures, the slope of the ordinary least squares regression or Measure 1 on Measure 2 was used to examine potential regression dilution bias [11].

To address the main objective of the study, multivariate logistic regression models were used to explore the predictive ability of serum urate measures on gout incidence. The binary outcome variable was gout incidence (gout/no gout) and the predictor variable was one of the following four measures of urate exposure, reflecting single and serial testing:

1. First serum urate measure (i.e. Measure 1)
2. Second serum urate measure (i.e. Measure 2)
3. Average of Measure 1 and Measure 2
4. Highest of Measure 1 and Measure 2

The cohort, baseline age, and sex were force-entered into all models as covariates, while baseline BMI and renal function were included only if *P* ≥ 0.10.

To provide a comparison of the predictive ability between the different models, receiver operator characteristic (ROC) curves were generated from each of the logistic models. ROC curves further from the diagonal line (representing a non-discriminating model) corresponded to a model that was better at discriminating between positive and negative gout incident cases. The area under the ROC curves (AUC) for each model were calculated and the concordance statistics (*c*-statistic) were reported to provide a performance metric for each ROC curve. The AUC *c*-statistic ranges from 0.5 (no discrimination) to 1 (perfect discrimination). Differences between *c*-statistics for each model were considered significant at *P* < 0.05 if no overlap was observed between the 95% confidence intervals. This comparison method was selected due to the large sample size and the high correlation between the ROC curves which may have resulted in significant but biologically trivial differences between the models if more formal tests were conducted (i.e. the method of DeLong et al.). Sensitivity, specificity, positive predictive value (PPV), negative predictive value (NPV), and accuracy were calculated for each model using the following pre-specified cut-points: 357 μmol/L (6.0 mg/dL), 416 μmol/L (7.0 mg/dL), and 476 μmol/L (8.0 mg/dL), to provide a comparison between models across a small subset of clinically relevant values. The above analyses were also undertaken separately for men and women, and for women according to age (< 51 years and ≥ 51 years) to reflect the influence of menopause on serum urate levels [12]. A sensitivity analysis was undertaken using only data from ARIC and CARDIA (FHS excluded) due to differences in the pattern of serum urate over time between the cohorts.

As a further sensitivity analysis, to explore the heterogeneity between data from the three included studies, traditional study-level meta-analyses were performed for each model to explore the difference in AUC c-statistics. Chi^2^, I^2^, and associated P values were computed for each meta-analysis.

Analyses were undertaken in SPSS (v 25), RevMan (v 5.4), and RStudio (v 1.3.959).

## Results

### Participant characteristics

A total of 16,017 participants were included across the three cohorts. ARIC contributed the largest number of participants (n = 10,091), followed by FHS (n = 3,099) and CARDIA (n = 2,827). Detailed demographic and clinical characteristics for the included participants are shown in S1 Table. Overall, 55.7% of participants were female, and 79.5% were European, and the remaining 20.5% African American. The mean age of participants at Measure 1 was 49 years. The mean (SD) time between Measure 1 and Measure 2 was 3.5 (0.9) years, and between Measure 2 and the Gout Assessment visit was 5.8 (0.6) years. The total mean follow-up time was 9.3 (0.7) years.

### Serum urate between Measure 1 and Measure 2

Differences in the change in serum urate over time were observed between the three cohorts, with ARIC and CARDIA demonstrating increases, and FHS demonstrating a decrease. S2 Table presents the descriptive statistics for serum urate for the first measure, the second measure, the average of the two measures and the highest of the two measures for each of the three cohorts. After adjusting for cohort, there was a significant overall increase in mean serum urate over time (322 μmol/L (5.42 mg/dL) at Measure 1 vs. 340 μmol/L (5.71 mg/dL) at Measure 2, *P* < 0.001). Examination of the correlation between the two urate measures demonstrated a high correlation (overall Pearson’s *r* = 0.814, *P* < 0.001). The ordinary least squares regression coefficient of first vs. second measurement was 1.18, less than the 1.2 rule of thumb suggesting significant regression dilution bias.

### Prediction of incident gout for each serum urate testing model

**Fig 2:**
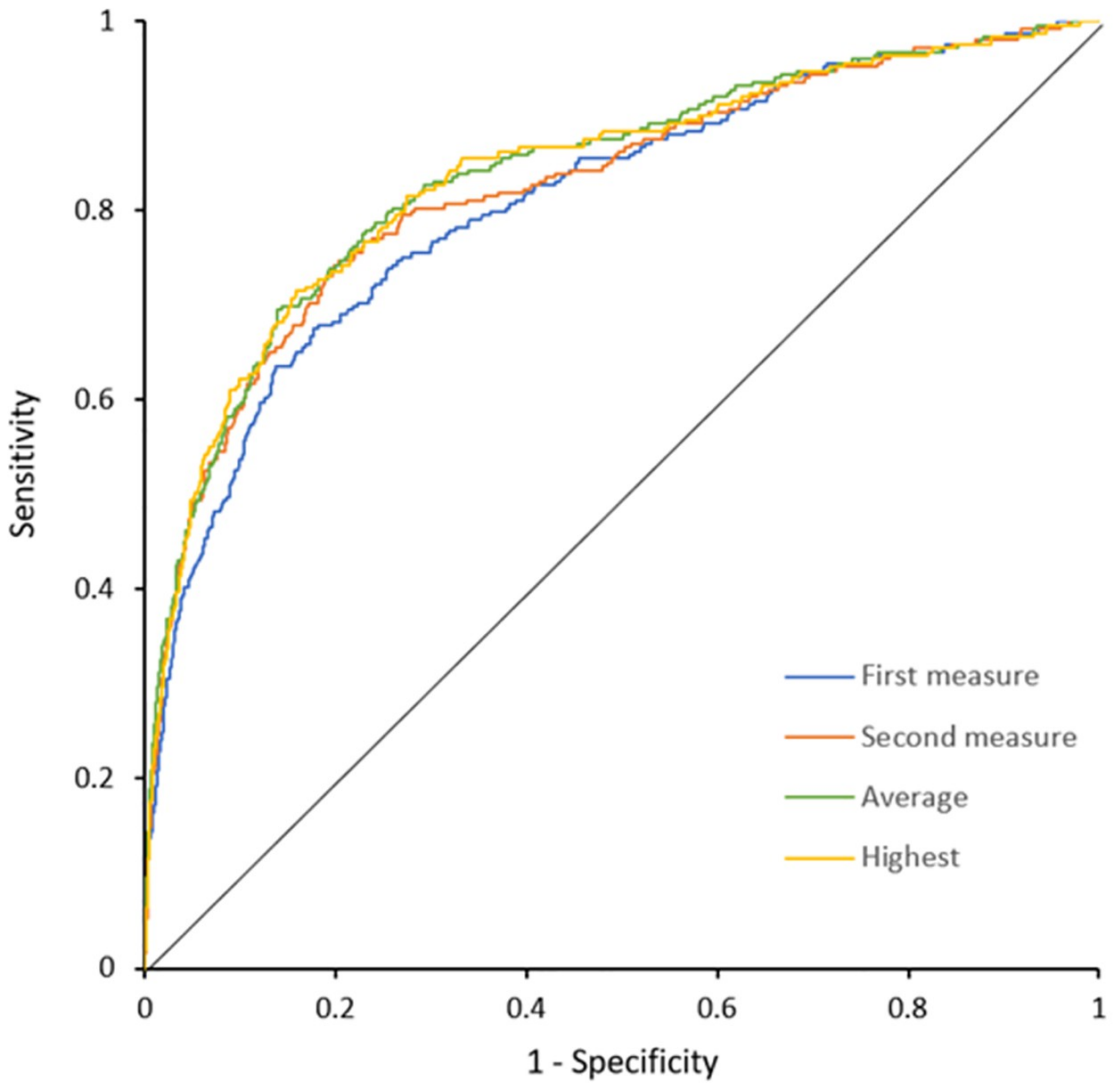
ROC curves showing discriminative ability of each model in predicting incident gout.

Incident gout occurred in 249 (1.6%) participants between urate Measure 2 and the Gout Assessment visit. The ROC curve analysis showed no significant difference in the predictive ability of incident gout between the different measurements of serum urate, evident by the substantial overlap of the confidence intervals (Table 1, Fig 2). The AUC *c*-statistics demonstrated high discrimination between participants with and without incident gout across all four urate measurements, ranging from 0.81 (95% CI: 0.78, 0.84) to 0.84 (95% CI: 0.78, 0.84) (Table 1).

**Table 1:** Predictive value of serum urate measures for gout incidence.

Measurement		ROC curve analysis		Predictive cut points					
		AUC (95% CI)	*P*	Cut point	Sensitivity	Specificity	PPV	NPV	Accuracy
1	First measure	0.81 (0.78, 0.84)	<0.001	357 μmol/L (6.0 mg/dL)	75.2% (69.3%, 80.5%)	68.3% (67.5%, 69.0%)	3.5% (3.3%, 3.8%)	99.5% (99.3%, 99.6%)	68.4% (67.6%, 69.1%)
				416 μmol/L (7.0 mg/dL)	57.6% (51.1%, 63.8%)	86.7% (86.1%, 87.2%)	6.3% (5.7%, 7.0%)	99.2% (99.1%, 99.4%)	86.2% (85.7%, 88.8%)
				476 μmol/L (8.0 mg/dL)	37.6% (31.5%, 43.9%)	95.2% (94.9%, 95.6%)	10.9% (9.3%, 12.7%)	99.0% (98.9%, 99.1%)	94.4% (94.0%, 94.7%)
2	Second measure	0.83 (0.80, 0.86)	<0.001	357 μmol/L (6.0 mg/dL)	72.5% (72.8%, 83.5%)	61.0% (60.3%, 91.8%)	3.0% (2.8%, 3.2%)	99.5% (99.3%, 99.6%)	61.3% (60.5%, 62.0%)
				416 μmol/L (7.0 mg/dL)	66.0% (60.5%, 72.7%)	80.0% (79.4%, 80.7%)	4.9% (4.5%, 5.4%)	99.4% (99.4%, 99.5%)	79.8% (79.2%, 80.5%)
				476 μmol/L (8.0 mg/dL)	50.6% (44.2%, 57.0%)	91.8% (91.4%, 92.3%)	8.8% (7.8%, 9.9%)	99.2% (99.1%, 99.3%)	91.2% (90.8%, 91.6%)
3	Average of both measures	0.84 (0.81. 0.87)	<0.001	357 μmol/L (6.0 mg/dL)	79.1% (73.5%, 84.0%)	64.3% (63.6%, 65.0%)	3.4% (3.2%, 3.6%)	99.5% (99.4%, 99.6%)	64.5% (63.8%, 65.3%)
				416 μmol/L (7.0 mg/dL)	65.9% (59.6%, 71.7%)	83.7% (83.1%, 84.3%)	6.0% (5.5%, 6.6%)	99.4% (99.2%, 99.5%)	83.4% (82.8%, 84.0%)
				476 μmol/L (8.0 mg/dL)	45.8% (39.5%, 52.2%)	94.6% (94.2%, 94.9%)	11.7% (10.3%, 13.4%)	99.1% (99.0%, 99.2%)	93.8% (93.4%, 94.2%)
4	Highest of both measures	0.84 (0.81, 0.87)	<0.001	357 μmol/L (6.0 mg/dL)	82.8% (77.5%, 87.3%)	55.8% (55.0%, 86.6%)	2.8% (2.7%, 3.0%)	99.5% (99.4%, 99.6%)	56.2% (55.4%, 57.0%)
				416 μmol/L (7.0 mg/dL)	71.3% (65.2%, 76.9%)	76.7% (76.0%, 77.3%)	4.5% (4.2%, 4.9%)	99.4% (99.3%, 99.5%)	76.6% (75.9%, 77.2%)
				476 μmol/L (8.0 mg/dL)	56.7% (50.3%, 63.0%)	89.9% (89.4%, 90.4%)	8.1% (7.2%, 9.0%)	99.3% (99.1%, 99.4%)	89.4% (88.9%, 89.9%)

All models were adjusted for sex, age, and cohort. BMI and renal function did not significantly contribute to the models (P>0.10) and were excluded as covariates. ROC = receiver operator characteristic; AUC = area under the curve; CI = confidence interval; PPV = positive predictive value; NPV = negative predictive value. Accuracy = defined as the number of true positive plus true negatives divided by the total number of participants.

Sensitivity, specificity, NPV, PPV, and accuracy values were similar across all models (Table 1). The sensitivity increased with the lowest cut point (i.e., 357 μmol/L (6.0 mg/dL)), ranging from 72.5% to 82.8%, while specificity increased with the highest cut point (i.e., 476 μmol/L (8.0 mg/dL)), ranging from 89.9% to 95.2% across the four urate measurement models. The overall accuracy for each model was highest for the 476 μmol/L (8.0 mg/dL) cut point, ranging from 89.4% to 94.4% across the different models of urate measurement.

Additional analyses by gender showed similar findings for both men and women in which the predictive ability of incident gout did not significantly differ across the four models of urate measurement (S3 and S4 Tables, S2 Fig). The sensitivity of each urate model was consistently higher for men compared to women across all urate measurement models. A further analysis for women aged < 51 years and ≥ 51 years, showed a higher sensitivity for the prediction of incident gout among the older age group (S5 and S6 Tables, S2 Fig).

Results from the sensitivity analysis using data from ARIC and CARDIA (FHS excluded) are shown in S7 Table. The findings are consistent with the primary analysis demonstrating the same overall pattern across all models, with the highest accuracy observed for the highest urate cut point. A lack of statistically significant heterogeneity was also demonstrated across the four measurement models based on the results of the cohort-level meta-analyses shown in S8 Table.

## Discussion

This analysis of individual participant data demonstrates a small increase in serum urate levels over 3.5 years. However, repeat testing of serum urate over time does not improve the predictive ability of a single urate test for incident gout.

Despite the small increase in urate over time, the change appears to be not clinically relevant when considering the predictive ability of repeated urate measurements for incident gout. The association between urate concentrations and gout has been well established, and measuring urate concentration is crucial for diagnosing and monitoring of patients with, and at risk of, gout. However, the current analysis suggests that repeated measures of urate do not improve the sensitivity or accuracy for predicting incident gout compared to single measures of urate over a period of 3.5 years. Whether a repeat serum urate measurement after a longer period would improve prediction cannot be determined from these data.

The well-established linear relationship between increasing serum urate concentrations and incident gout [5] was also demonstrated in the current study with higher urate levels corresponding to greater sensitivity for predicting incident gout, regardless of the urate measurement model (i.e. repeat testing vs. single testing). This pattern was similar for both men and women, with the sensitivity of serum urate measurement for gout incidence being higher among men and post-menopausal women.

Some limitations should be acknowledged. The variation in serum urate concentration between the three cohorts may be due to the method of urate measurement used. However, all analyses adjusted for cohort to account for potential between-cohort heterogeneity. Additionally, participants with gout were identified based on a self-reported diagnosis of gout. However, compared to the gold standard monosodium urate crystal identification, a definition of self-reported gout performs well [10]. It should also be recognised that both diurnal and seasonal variations in serum urate have been reported with slightly higher concentrations in the morning and in summer [13, 14]. Due to participant confidentiality, the time and date of urate testing was not available for extraction from the cohort databases. However, given the large number of individual patient data included in this analysis, it is unlikely that there would be systemic bias that would influence the results. Furthermore, in order to determine the predictive ability of repeat testing of serum urate on gout incidence, participants were excluded if they did not have paired serum urate measures 3–5 years apart and if they developed gout prior to or between these urate measures. This resulted in a smaller proportion who developed incident gout between the second measure and the gout assessment visit. Although this exclusion criteria were necessary to address the research question, it should be acknowledged that this may have resulted in an under-representation of gout incidence, and may limit generalizability of these results to person’s with an increased risk of gout. Finally, the low PPVs observed in the current analysis were a function of the low background incidence of gout in the included cohorts (1.6%). For example, increasing the prevalence of gout to 4.7% (to reflect the background prevalence of gout when including participants who developed gout prior to the second measure of urate) would have increased the PPV from 3.5% to 10.4% for the predictive value of the first serum urate measure at the 357 μmol/L (6.0 mg/dL) cut point [15].

## Conclusions

In conclusion, this study shows that repeat testing of serum urate 3.5 years apart is not superior to a single measurement with regard to predictive performance for gout incidence. These results may inform the design of longitudinal studies of incident gout. In conjunction with other clinical, family history and laboratory variables shown to predict gout incidence, these findings may also inform clinical practice when providing advice to individuals about their risk of developing gout.

## Supporting information

S1 Fig Flow chart of participants included from the three cohorts. (DOCX)

S2 Fig ROC curves showing the ability of each model to predict incident gout in men (A), women (B), women aged < 51 years (C), and women aged > 51 years (D). (DOCX)

S1 Table Characteristics of participants excluded vs included in the analysis. (DOCX)

S2 Table Serum urate between Measure 1 and Measure 2. (DOCX)

S3 Table Predictive value of serum urate measures for gout incidence for men. (DOCX)

S4 Table Predictive value of serum urate measures for gout incidence for women. (DOCX)

S5 Table Predictive value of serum urate measures for gout incidence for women < 51 years (n = 5075). (DOCX)

S6 Table Predictive value of serum urate measures for gout incidence for women >51 years (n = 3839). (DOCX)

S7 Table Predictive value of serum urate measures for gout incidence using ARIC and CARDIA cohorts (FHS excluded). (DOCX)

S8 Table Heterogeneity statistics for difference in AUCs between cohorts. (DOCX)
