# Supplementary figures and images for: Is repeat serum urate testing superior to a single test to predict incident gout over time?
Source: PLoS One. 2022 Feb 1;17(2):e0263175. doi: 10.1371/journal.pone.0263175 (PMC8806054; doi:10.1371/journal.pone.0263175)

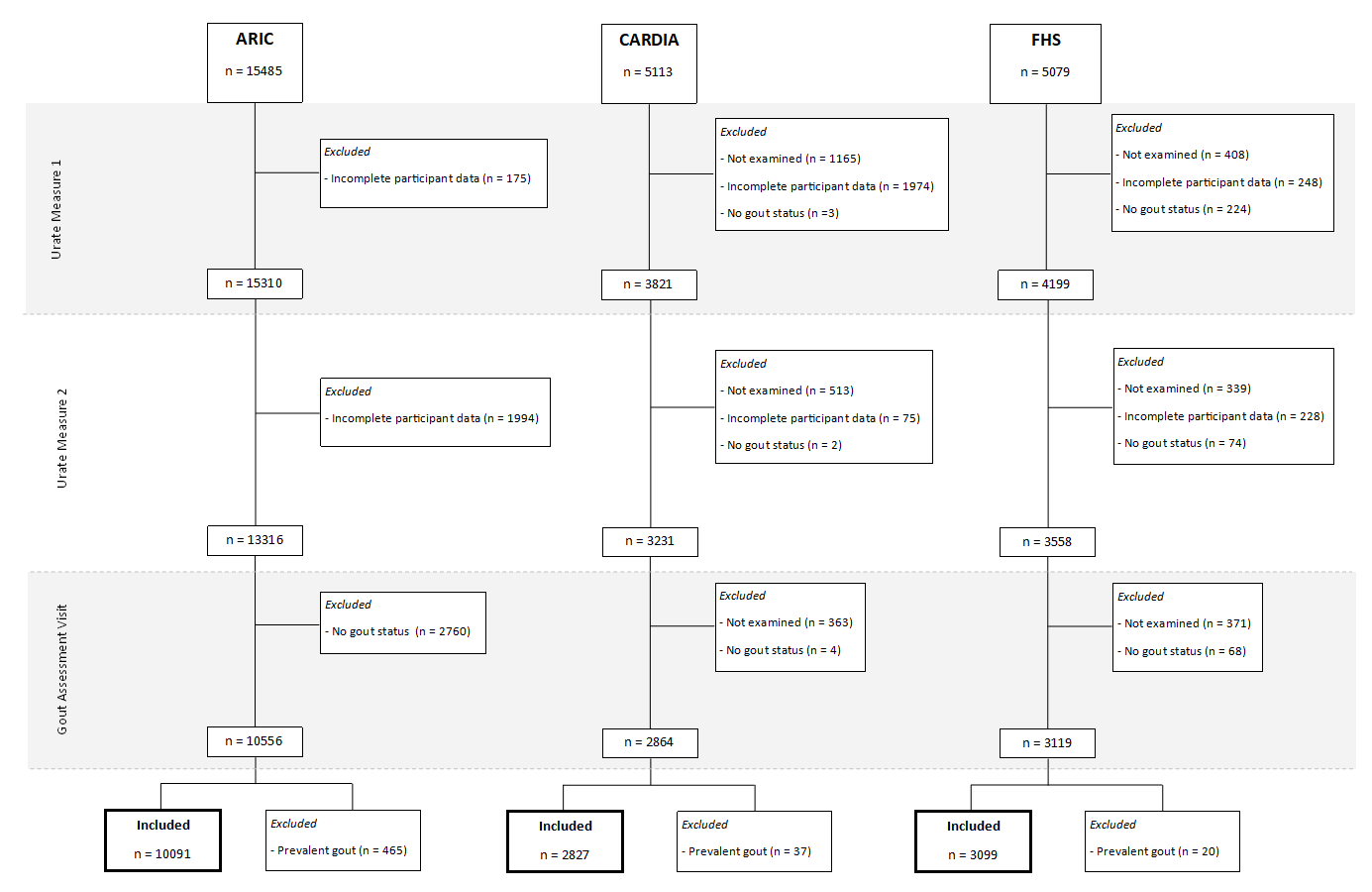
***S1 Figure****. Flow chart of participants included from the three cohorts.*

Supplement: S1 Fig — (DOCX) [file pone.0263175.s001.docx]
