# Supplementary material for: Is repeat serum urate testing superior to a single test to predict incident gout over time?
Source: PLoS One. 2022 Feb 1;17(2):e0263175. doi: 10.1371/journal.pone.0263175 (PMC8806054; doi:10.1371/journal.pone.0263175)
Supplement: S2 Fig — (DOCX) [file pone.0263175.s002.docx]

*
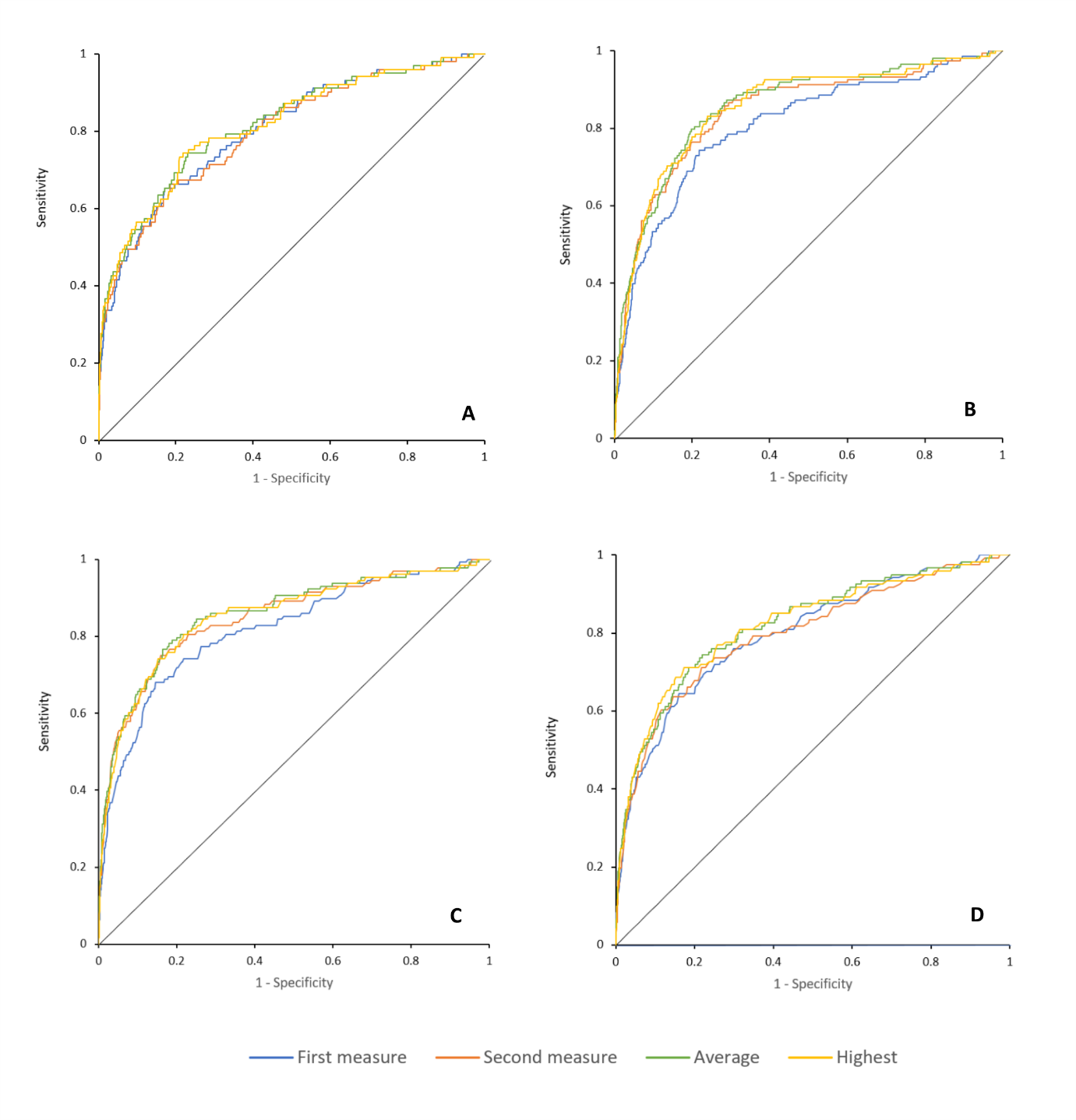
*

***S2 Figure.*** *ROC curves showing the ability of each model to predict incident gout in men (A), women (B), women aged < 51 years (C), and women aged > 51 years (D).*
