# Supplementary material for: Is repeat serum urate testing superior to a single test to predict incident gout over time?
Source: PLoS One. 2022 Feb 1;17(2):e0263175. doi: 10.1371/journal.pone.0263175 (PMC8806054; doi:10.1371/journal.pone.0263175)
Supplement: S1 Table — (DOCX) [file pone.0263175.s003.docx]

| **S1 Table.** Characteristics of participants excluded vs included in the analysis^a^ | | | | | | | | | |
| --- | --- | --- | --- | --- | --- | --- | --- | --- | --- |
|  | | **ARIC** | | **CARDIA** | | **FHS** | | **Total** | |
|  | | **Excluded** | **Included** | **Excluded** | **Included** | **Excluded** | **Included** | **Excluded** | **Included** |
| N | | 465 | 10091 | 37 | 2827 | 20 | 3099 | 522 | 16017 |
| Developed gout at/prior to Measure 1 | | 397 (85.4%) | NA | 17 (45.9%) | NA | 9 (45.0%) | NA | 423 (81.0%) | NA |
| Developed gout between Measure 1 and Measure 2 | | 68 (14.6%) | NA | 20 (54.1%) | NA | 11 (55.0%) | NA | 99 (19.0%) | NA |
| Incident gout between Measure 2 and Gout Assessment | | NA | 173 (1.7%) | NA | 36 (1.3%) | NA | 40 (1.3%) | NA | 249 (1.6%) |
| Sex, n (%)  Female  Male | | 140 (30.1%)  325 (69.9%) | 5666 (56.1%)  4425 (43.9%) | 11 (29.7%)  26 (70.3%) | 1573 (55.6%)  1254 (44.4%) | 0 (0.0%)  20 (100%) | 1845 (54.0%)  1254 (46.0%) | 151 (28.9%)  371 (71.1%) | 8914 (55.7%)  7103 (44.3%) |
| Ethnicity, n (%)  European  African American | | 357 (76.8%)  108 (23.2%) | 8053 (79.8%)  2038 (20.2%) | 17 (45.9%)  20 (54.1%) | 1583 (56.0%)  1244 (44.0%) | 20 (100%)  0 (0%) | 3099 (100%)  0 (0%) | 394 (75.5%)  128 (24.5%) | 12735 (79.5%)  3282 (20.5%) |
| Measure 1 | Age, years | 55.6 (5.7) | 53.9 (5.7) | 36.7 (3.0) | 35.2 (3.6) | 48.3 (7.4) | 45.7 (8.4) | 54.0 (7.5) | 49.0 (9.3) |
|  | Urate, µmol/L | 446 (113) | 351 (89) | 387 (119) | 280 (83) | 399 (89) | 268 (71) | 440 (113) | 321 (89) |
|  | Urate, mg/dL | 7.5 (1.9) | 5.9 (1.5) | 6.5 (2.0) | 4.7 (1.4) | 6.7 (1.5) | 4.5 (1.2) | 7.4 (1.9) | 5.4 (1.5) |
|  | BMI, kg/m^2^ | 30.0 (5.4) | 27.4 (5.1) | 29.1 (6.6) | 27.2 (5.9) | 28.2 (4.3) | 25.8 (4.0) | 29.9 (5.5) | 27.0 (5.1) |
|  | Creatinine, μmol/L | 107.9 (53.6) | 96.5 (24.6) | 87.4 (14.4) | 82.9 (17.2) | NR | NR | 106.4 (52.0) | 93.5 (23.8) |
|  | Creatinine, mg/dl | 1.22 (0.61) | 1.09 (0.28) | 0.99 (0.16) | 0.94 (0.20) | NR | NR | 1.20 (0.59) | 1.06 (0.27) |
|  | eGFR | 66.8 (13.0) | 69.4 (11.9) | 98.5 (14.5) | 98.1 (16.0) | NR | NR | 69.1 (15.5) | 75.7 (17.5) |
| Measure 2 | Age, years | 58.5 (5.7) | 56.9 (5.7) | 41.8 (3.0) | 40.3 (3.6) | 52.2 (7.4) | 49.5 (8.4) | 57.1 (7.1) | 52.5 (8.7) |
|  | Urate, µmol/L | 476 (125) | 381 (89) | 375 (107) | 286 (89) | 369 (77) | 262 (59) | 464 (125) | 339 (101) |
|  | Urate, mg/dL | 8.0 (2.1) | 6.4 (1.5) | 6.3 (1.8) | 4.8 (1.5) | 6.2 (1.3) | 4.4 (1.0) | 7.8 (2.1) | 5.7 (1.7) |
|  | BMI, kg/m^2^ | 30.2 (5.6) | 27.8 (5.2) | 30.0 (6.4) | 28.5 (6.4) | 27.6 (3.4) | 26.0 (4.0) | 30.1 (5.6) | 27.5 (5.3) |
|  | Creatinine, μmol/L | 113.0 (42.2) | 100.7 (27.6) | 90.2 (13.6) | 87.0 (27.1) | NR | NR | 111.3 (41.2) | 97.7 (28.1) |
|  | Creatinine, mg/dl | 1.27 (0.48) | 1.14 (0.31) | 1.04 (0.15) | 1.00 (0.31) | NR | NR | 1.26 (0.47) | 1.10 (0.32) |
|  | eGFR | 62.1 (13.4) | 64.7 (11.2) | 91.8 (13.2) | 90.2 (15.4) | NR | NR | 98.2 (17.3) | 96.5 (17.5) |
| Years between Measures | | 2.9 (0.4) | 2.9 (0.4) | 5.1 (0.4) | 5.1 (0.5) | 3.9 (0.4) | 3.8 (0.6) | 3.1 (0.7) | 3.5 (0.9) |
| Years between Measure 1 and Gout Assessment | | 8.9 (0.5) | 8.9 (0.5) | 10.2 (0.4) | 10.1 (0.5) | 9.9 (0.4) | 9.8 (0.5) | 9.0 (0.6) | 9.3 (0.7) |
| Years between Measure 2 and Gout Assessment | | 6.0 (0.5) | 6.0 (0.5) | 5.1 (0.5) | 5.0 (0.5) | 6.0 (0.5) | 6.0 (0.4) | 5.9 (0.5) | 5.8 (0.6) |
| ^a^Excluded participants had prevalent gout prior to Measure 2. Included participants had no prior recording of gout before Measure 2. NA = Not applicable; NR = Not reported. Data presented as mean (SD), unless otherwise indicated. | | | | | | | | | |
