# Supplementary material for: Is repeat serum urate testing superior to a single test to predict incident gout over time?
Source: PLoS One. 2022 Feb 1;17(2):e0263175. doi: 10.1371/journal.pone.0263175 (PMC8806054; doi:10.1371/journal.pone.0263175)
Supplement: S2 Table — (DOCX) [file pone.0263175.s004.docx]

| **S2 Table.** Serum urate between Measure 1 and Measure 2 | | | | | | | | | |
| --- | --- | --- | --- | --- | --- | --- | --- | --- | --- |
|  | **n** | **Serum urate, mean (SD)** | | | | | **Years between Measure 1 and Measure 2, mean (SD)** | **Correlation between Measure 1 and Measure 2** | |
|  |  | **Unit** | **First measure** | **Second measure** | **Average of both measures** | **Highest of both measures** |  | **Pearson’s *r*** | ***P*** |
| ARIC | 10091 | **µmol/L** | 351 (86) | 378 (90) | 365 (83) | 389 (90) | 174 (23) | 0.778 | <0.001 |
|  |  | **mg/dL** | 5.90 (1.45) | 6.36 (1.51) | 6.13 (1.40) | 6.54 (1.52) | 2.92 (0.39) |  |  |
| CARDIA | 2827 | **µmol/L** | 282 (81) | 287 (89) | 284 (81) | 305 (87) | 303 (29) | 0.811 | <0.001 |
|  |  | **mg/dL** | 4.74 (1.36) | 4.83 (1.49) | 4.78 (1.36) | 5.12 (1.46) | 5.09 (0.49) |  |  |
| FHS | 3099 | **µmol/L** | 267 (81) | 262 (61) | 264 (59) | 284 (65) | 228 (34) | 0.672 | <0.001 |
|  |  | **mg/dL** | 4.49 (1.16) | 4.40 (1.03) | 4.44 (1.00) | 4.78 (1.09) | 3.84 (0.57) |  |  |
| Overall | 16071 | **µmol/L** | 267 (69) | 340 (99) | 331 (90) | 354 (97) | 207 (56) | 0.814 | <0.001 |
|  |  | **mg/dL** | 5.42 (1.52) | 5.71 (1.67) | 5.57 (1.52) | 5.95 (1.63) | 3.48 (0.94) |  |  |
