# Supplementary material for: Is repeat serum urate testing superior to a single test to predict incident gout over time?
Source: PLoS One. 2022 Feb 1;17(2):e0263175. doi: 10.1371/journal.pone.0263175 (PMC8806054; doi:10.1371/journal.pone.0263175)
Supplement: S3 Table — (DOCX) [file pone.0263175.s005.docx]

| **S3 Table.** Predictive value of serum urate measures for gout incidence for men | | | | | | | | | |  |
| --- | --- | --- | --- | --- | --- | --- | --- | --- | --- | --- |
| **Measurement** | | **ROC curve analysis** | | **Predictive cut points** | | | | | | |
|  |  | **AUC (95% CI)** | **P** | **Cut point** | **Sensitivity** | **Specificity** | **PPV** | **NPV** | **Accuracy** | |
| 1 | First measure | 0.81 (0.77, 0.85) | **<0.001** | 357 µmol/L (6.0 mg/dL) | 86.2% (79.5%, 91.4%) | 50.1% (48.9%, 51.3%) | 3.5% (3.3%, 3.7%) | 99.4% (99.1%, 99.6%) | 50.9% (49.7%, 52.0%) | |
|  |  |  |  | 416 µmol/L (7.0 mg/dL) | 66.0% (57.7%, 73.6%) | 77.8% (76.8%, 78.8%) | 5.9% (5.3%, 6.7%) | 99.1% (98.9%, 99.3%) | 77.6% (76.6%, 78.6%) | |
|  |  |  |  | 476 µmol/L (8.0 mg/dL) | 42.2% (34.1%, 50.6%) | 92.1% (91.4%, 92.7%) | 10.2% (8.4%, 12.2%) | 98.7% (98.5%, 98.9%) | 91.1% (90.4%, 91.7%) | |
| 2 | Second measure | 0.84 (0.81, 0.88) | **<0.001** | 357 µmol/L (6.0 mg/dL) | 88.3% (81.9%, 93.0%) | 43.5% (42.3%, 44.7%) | 3.2% (3.0%, 3.4%) | 99.4% (99.1%, 99.6%) | 44.4% (43.3%, 45.6%) | |
|  |  |  |  | 416 µmol/L (7.0 mg/dL) | 78.9% (71.4%, 85.2%) | 68.1% (67.0%, 69.2%) | 5.0% (4.6%, 5.4%) | 99.4% (99.1%, 99.5%) | 68.3% (67.2%, 69.4%) | |
|  |  |  |  | 476 µmol/L (8.0 mg/dL) | 59.3% (50.9%, 67.4%) | 86.0% (85.1%, 86.8%) | 8.0% (7.0%, 9.2%) | 99.0% (98.8%, 99.2%) | 85.4% (84.6%, 86.2%) | |
| 3 | Average of both measures | 0.85 (0.82, 0.89) | **<0.001** | 357 µmol/L (6.0 mg/dL) | 88.5% (82.3%, 93.2%) | 45.3% (44.2%, 46.5%) | 3.3% (3.1%, 3.5%) | 99.5% (99.2%, 99.7%) | 46.2% (45.1%, 47.4%) | |
|  |  |  |  | 416 µmol/L (7.0 mg/dL) | 77.0% (69.4%, 83.5%) | 72.9% (71.9%, 74.0%) | 5.7% (5.2%, 6.3%) | 99.3% (99.1%, 99.5%) | 73.0% (72.0%, 74.0%) | |
|  |  |  |  | 476 µmol/L (8.0 mg/dL) | 50.7% (42.3%, 59.0%) | 90.9% (90.2%, 91.5%) | 10.6% (9.0%, 12.3%) | 98.9% (98.7%, 99.0%) | 90.0% (89.3%, 90.7%) | |
| 4 | Highest of both measures | 0.85 (0.82, 0.89) | **<0.001** | 357 µmol/L (6.0 mg/dL) | 92.5% (87.0%, 96.2%) | 36.4% (35.3%, 37.5%) | 3.0% (2.9%, 3.1%) | 99.6% (99.2%, 99.8%) | 37.6% (36.4%, 38.7%) | |
|  |  |  |  | 416 µmol/L (7.0 mg/dL) | 82.3% (75.2%, 88.1%) | 62.9% (91.8%, 64.1%) | 4.5% (4.2%, 4.9%) | 99.4% (99.2% (99.6%) | 63.3% (62.2%, 64.5%) | |
|  |  |  |  | 476 µmol/L (8.0 mg/dL) | 65.3% (57.0%, 73.0%) | 83.5% (82.6%, 84.3%) | 7.7% (6.8%, 8.7%) | 99.1% (98.9%, 99.3%) | 83.1% (82.2%, 84.0%) | |
| All models were adjusted for age, and cohort. BMI and renal function did not significantly contribute to the models (P>0.10) and were excluded as covariates. ROC = receiver operator characteristic; AUC = area under the curve; CI = confidence interval; PPV = positive predictive value; NPV = negative predictive value. Accuracy = defined as the number of true positive plus true negatives divided by the total number of participants. | | | | | | | | | | |
