# Supplementary material for: Is repeat serum urate testing superior to a single test to predict incident gout over time?
Source: PLoS One. 2022 Feb 1;17(2):e0263175. doi: 10.1371/journal.pone.0263175 (PMC8806054; doi:10.1371/journal.pone.0263175)
Supplement: S4 Table — (DOCX) [file pone.0263175.s006.docx]

| **S4 Table.** Predictive value of serum urate measures for gout incidence for women | | | | | | | | | |  |
| --- | --- | --- | --- | --- | --- | --- | --- | --- | --- | --- |
| **Measurement** | | **ROC curve analysis** | | **Predictive cut points** | | | | | | |
|  |  | **AUC (95% CI)** | **P** | **Cut point** | **Sensitivity** | **Specificity** | **PPV** | **NPV** | **Accuracy** | |
| 1 | First measure | 0.80 (0.76, 0.85) | **<0.001** | 357 µmol/L (6.0 mg/dL) | 58.8% (48.3%, 68.7%) | 82.5% (81.7%, 83.3%) | 3.6% (3.0%, 4.2%) | 99.5% (99.3%, 99.6%) | 82.3% (81.5%, 83.1%) | |
|  |  |  |  | 416 µmol/L (7.0 mg/dL) | 44.3% (24.2%, 54.8%) | 93.7% (93.1%, 94.2%) | 7.2% (5.7%, 8.9%) | 99.4% (99.2%, 99.5%) | 93.1% (92.6%, 93.7%) | |
|  |  |  |  | 476 µmol/L (8.0 mg/dL) | 29.2% (20.3%, 39.3%) | 97.7% (97.4%, 98.0%) | 12.3% (9.1%, 16.4%) | 99.2% (99.1%, 99.3%) | 97.0% (96.6%, 97.3%) | |
| 2 | Second measure | 0.80 (0.75, 0.85) | **<0.001** | 357 µmol/L (6.0 mg/dL) | 63.5% (53.1%, 73.1%) | 74.8% (73.9%, 75.7%) | 2.7% (2.3%, 3.1%) | 99.5% (99.3%, 99.6%) | 74.7% (73.8%, 75.6%) | |
|  |  |  |  | 416 µmol/L (7.0 mg/dL) | 47.9% (37.6%, 58.4%) | 89.5% (88.8%, 90.1%) | 4.7% (3.8%, 5.8%) | 99.4% (99.2%, 99.5%) | 89.0% (88.4%, 89.7%) | |
|  |  |  |  | 476 µmol/L (8.0 mg/dL) | 35.4% (25.9%, 45.8%) | 96.1% (95.6%, 96.5%) | 8.9% (6.8%, 11.5%) | 99.3% (99.2%, 99.4%) | 95.4% (94.9%, 95.8%) | |
| 3 | Average of both measures | 0.82 (0.77, 0.86) | **<0.001** | 357 µmol/L (6.0 mg/dL) | 63.5% (52.2%, 74.5%) | 79.2% (78.4%, 80.1%) | 3.5% (3.0%, 4.0%) | 99.5% (99.4%, 99.6%) | 79.1% (78.2%, 79.9%) | |
|  |  |  |  | 416 µmol/L (7.0 mg/dL) | 49.5% (39.4%, 59.6%) | 92.1% (91.6%, 92.7%) | 6.7% (5.5%, 8.2%) | 99.4% (99.2%, 99.5%) | 91.7% (91.1%, 92.2%) | |
|  |  |  |  | 476 µmol/L (8.0 mg/dL) | 38.6% (29.1%, 48.8%) | 97.5% (97.1%, 97.8%) | 14.9% (11.7%, 18.8%) | 99.3% (99.2%, 99.4%) | 96.8% (96.4%, 97.2%) | |
| 4 | Highest of both measures | 0.81 (0.77, 0.86) | **<0.001** | 357 µmol/L (6.0 mg/dL) | 67.4% (57.0%, 76.6%) | 71.1% (70.1%, 72.0%) | 2.5% (2.1%, 2.8%) | 99.5% (99.3%, 99.6%) | 71.1% (70.1%, 72.0%) | |
|  |  |  |  | 416 µmol/L (7.0 mg/dL) | 53.7% (43.2%, 64.0%) | 87.5% (86.8%, 88.2%) | 4.4% (3.7%, 5.3%) | 99.4% (99.3%, 99.5%) | 87.1% (86.4%, 87.8%) | |
|  |  |  |  | 476 µmol/L (8.0 mg/dL) | 42.1% (32.0%, 52.7%) | 95.0% (94.5%, 95.5%) | 8.3% (6.6%, 10.5%) | 99.4% (99.2%, 99.5%) | 94.4% (94.0%, 94.9%) | |
| All models were adjusted for age, and cohort. BMI and renal function did not significantly contribute to the models (P>0.10) and were excluded as covariates. ROC = receiver operator characteristic; AUC = area under the curve; CI = confidence interval; PPV = positive predictive value; NPV = negative predictive value. Accuracy = defined as the number of true positive plus true negatives divided by the total number of participants. | | | | | | | | | | |
