# Supplementary material for: Is repeat serum urate testing superior to a single test to predict incident gout over time?
Source: PLoS One. 2022 Feb 1;17(2):e0263175. doi: 10.1371/journal.pone.0263175 (PMC8806054; doi:10.1371/journal.pone.0263175)
Supplement: S5 Table — (DOCX) [file pone.0263175.s007.docx]

| **S5 Table.** Predictive value of serum urate measures for gout incidence for women < 51 years (n = 5075) | | | | | | | | | |  |
| --- | --- | --- | --- | --- | --- | --- | --- | --- | --- | --- |
| **Measurement** | | **ROC curve analysis** | | **Predictive cut points** | | | | | | |
|  |  | **AUC (95% CI)** | **P** | **Cut point** | **Sensitivity** | **Specificity** | **PPV** | **NPV** | **Accuracy** | |
| 1 | First measure | 0.79 (0.72, 0.86) | **<0.001** | 357 µmol/L (6.0 mg/dL) | 44.2% (29.1%, 60.1%) | 89.4% (88.5%, 90.3%) | 3.5% (2.5%, 5.9%) | 99.5% (99.3%, 99.6%) | 89.0% (88.1%, 89.9%) | |
|  |  |  |  | 416 µmol/L (7.0 mg/dL) | 32.6% (19.1%, 48.5%) | 96.5% (96.6%, 97.0%) | 7.4% (4.8%, 11.1%) | 99.4% (99.3%, 99.%) | 96.0% (95.4%, 96.5%) | |
|  |  |  |  | 476 µmol/L (8.0 mg/dL) | 20.9% (10.0%, 36.0%) | 98.9% (98.6%, 99.2%) | 14.1% (8.0%, 23.6%) | 99.3% (99.2%, 99.4%) | 98.2% (97.8%, 98.6%) | |
| 2 | Second measure | 0.81 (0.74, 0.88) | **<0.001** | 357 µmol/L (6.0 mg/dL) | 54.6% (68.9%, 69.6%) | 83.8% (82.8%, 94.8%) | 2.9% (2.2%, 3.7%) | 99.5% (99.4%, 99.7%) | 83.5% (82.5%, 84.6%) | |
|  |  |  |  | 416 µmol/L (7.0 mg/dL) | 43.2% (28.4%, 59.0%) | 93.9% (93.2%, 94.5%) | 5.8% (4.1%, 8.1%) | 99.5% (99.3%, 99.6%) | 93.4% (92.7%, 94.1%) | |
|  |  |  |  | 476 µmol/L (8.0 mg/dL) | 23.3% (11.8%, 38.6%) | 97.8% (97.3%, 98.2%) | 8.2% (4.8%, 13.7%) | 99.3% (99.2%, 99.4%) | 97.1% (96.6%, 97.6%) | |
| 3 | Average of both measures | 0.82 (0.75, 0.89) | **<0.001** | 357 µmol/L (6.0 mg/dL) | 53.3% (37.9%, 68.3%) | 87.4% (86.5%, 88.3%) | 3.7% (2.8%, 4.8%) | 99.5% (99.4%, 99.7%) | 87.1% (86.2%, 88.0%) | |
|  |  |  |  | 416 µmol/L (7.0 mg/dL) | 40.0% (25.7%, 55.7%) | 95.8% (95.2%, 96.4%) | 7.9% (5.5%, 11.1%) | 99.4% (99.3%, 99.6%) | 95.3% (94.7%, 95.9%) | |
|  |  |  |  | 476 µmol/L (8.0 mg/dL) | 28.9% (16.4%, 44.3%) | 98.8% (94.4%, 99.0%) | 17.1% (10.9%, 25.8%) | 99.4% (99.2%, 99.5%) | 98.1% (97.7%, 98.5%) | |
| 4 | Highest of both measures | 0.82 (0.75, 0.89) | **<0.001** | 357 µmol/L (6.0 mg/dL) | 56.8% (41.0%, 71.7%) | 80.7% (79.6%, 81.8%) | 2.5% (1.9%, 3.2%) | 99.5% (99.4%, 99.7%) | 80.5% (79.4%, 81.6%) | |
|  |  |  |  | 416 µmol/L (7.0 mg/dL) | 45.5% (30.4%, 61.2%) | 92.7% (92.0%, 93.4%) | 5.2% (3.7%, 7.1%) | 99.5% (99.3%, 99.6%) | 92.3% (91.5%, 93.0%) | |
|  |  |  |  | 476 µmol/L (8.0 mg/dL) | 30.2% (17.2%, 46.1%) | 97.2% (96.7%, 97.7%) | 8.5% (5.4%, 13.1%) | 99.4% (99.3%, 99.5%) | 96.7% (96.1%, 97.1%) | |
| All models were adjusted for cohort. BMI and renal function did not significantly contribute to the models (P>0.10) and were excluded as covariates. ROC = receiver operator characteristic; AUC = area under the curve; CI = confidence interval; PPV = positive predictive value; NPV = negative predictive value. Accuracy = defined as the number of true positive plus true negatives divided by the total number of participants. | | | | | | | | | | |
