# Supplementary material for: Is repeat serum urate testing superior to a single test to predict incident gout over time?
Source: PLoS One. 2022 Feb 1;17(2):e0263175. doi: 10.1371/journal.pone.0263175 (PMC8806054; doi:10.1371/journal.pone.0263175)
Supplement: S6 Table — (DOCX) [file pone.0263175.s008.docx]

| **S6 Table.** Predictive value of serum urate measures for gout incidence for women >51 years (n = 3839) | | | | | | | | | |  |
| --- | --- | --- | --- | --- | --- | --- | --- | --- | --- | --- |
| **Measurement** | | **ROC curve analysis** | | **Diagnostic cut points** | | | | | | |
|  |  | **AUC (95% CI)** | **P** | **Cut point** | **Sensitivity** | **Specificity** | **PPV** | **NPV** | **Accuracy** | |
| 1 | First measure | 0.80 (0.73, 0.87) | **<0.001** | 357 µmol/L (6.0 mg/dL) | 70.4% (56.4%. 82.0%) | 73.4% (72.0%, 74.8%) | 3.6% (3.1%, 4.3%) | 99.4% (99.1%, 99.6%) | 73.4% (72.0%, 74.8%) | |
|  |  |  |  | 416 µmol/L (7.0 mg/dL) | 53.7% (39.6%, 67.4%) | 90.0% (89.0%, 90.9%) | 7.1% (5.5%, 9.1%) | 99.3% (99.0%, 99.5%) | 89.4% (88.4%, 90.4%) | |
|  |  |  |  | 476 µmol/L (8.0 mg/dL) | 35.9% (23.1%, 50.2%) | 96.2% (95.5%, 96.8%) | 11.6% (8.1%, 16.3%) | 99.1% (98.9%, 99.2%) | 95.3% (94.6%, 96.0%) | |
| 2 | Second measure | 0.78 (0.71, 0.85) | **<0.001** | 357 µmol/L (6.0 mg/dL) | 72.2% (58.4%, 83.5%) | 62.9% (61.4%, 64.5%) | 2.7% (2.3%, 3.2%) | 99.4% (99.0%, 99.6%) | 63.1% (61.5%, 64.6%) | |
|  |  |  |  | 416 µmol/L (7.0 mg/dL) | 51.9% (37.6%. 66.0%) | 83.6% (82.4%, 84.8%) | 4.2% (3.2%, 5.4%) | 99.2% (99.0%, 99.4%) | 83.2% (82.0%, 84.4%) | |
|  |  |  |  | 476 µmol/L (8.0 mg/dL) | 44.2% (30.5%. 58.7%) | 93.7% (92.9%, 94.5%) | 8.8% (6.5%, 11.8%) | 99.2% (99.0%, 99.4%) | 93.0% (92.2%, 93.8%) | |
| 3 | Average of both measures | 0.81 (0.74, 0.88) | **<0.001** | 357 µmol/L (6.0 mg/dL) | 75.0% (61.6%, 85.6%) | 68.3% (66.8%, 69.8%) | 3.4% (2.9%, 4.0%) | 99.5% (99.2%, 99.7%) | 68.4% (66.9%, 69.9%) | |
|  |  |  |  | 416 µmol/L (7.0 mg/dL) | 57.1% (43.2%, 70.3%) | 87.2% (86.1%, 88.3%) | 6.2% (5.0%, 7.8%) | 99.3% (99.0%, 99.5%) | 86.8% (85.7%, 87.9%) | |
|  |  |  |  | 476 µmol/L (8.0 mg/dL) | 46.4% (33.0%, 60.3%) | 95.8% (95.1%, 96.4%) | 14.1% (10.6%, 18.4%) | 99.2% (99.0%, 99.4%) | 95.1% (94.3%, 95.7%) | |
| 4 | Highest of both measures | 0.80 (0.74, 0.87) | **<0.001** | 357 µmol/L (6.0 mg/dL) | 77.4% (63.8%, 87.7%) | 58.3% (56.7%, 59.9%) | 2.5% (2.2%, 2.9%) | 99.5% (99.1%, 99.7%) | 58.6% (57.0%, 60.2%) | |
|  |  |  |  | 416 µmol/L (7.0 mg/dL) | 60.8% (46.1%, 74.2%) | 80.5% (79.2%, 81.8%) | 4.0% (3.2%, 5.0%) | 99.4% (99.1%, 99.5%) | 80.3% (79.0%, 81.5%) | |
|  |  |  |  | 476 µmol/L (8.0 mg/dL) | 51.0% (36.6%, 65.3%) | 92.1% (91.2%, 92.9%) | 8.0% (6.1%, 10.4%) | 99.3% (99.1%, 99.5%) | 91.5% (90.6%, 92.4%) | |
| All models were adjusted for age, and cohort. BMI and renal function did not significantly contribute to the models (P>0.10) and were excluded as covariates. ROC = receiver operator characteristic; AUC = area under the curve; CI = confidence interval; PPV = positive predictive value; NPV = negative predictive value. Accuracy = defined as the number of true positive plus true negatives divided by the total number of participants. | | | | | | | | | | |
