# Supplementary material for: Is repeat serum urate testing superior to a single test to predict incident gout over time?
Source: PLoS One. 2022 Feb 1;17(2):e0263175. doi: 10.1371/journal.pone.0263175 (PMC8806054; doi:10.1371/journal.pone.0263175)
Supplement: S7 Table — (DOCX) [file pone.0263175.s009.docx]

| **S7 Table.** Predictive value of serum urate measures for gout incidence using ARIC and CARDIA cohorts (FHS excluded) | | | | | | | | | |  |
| --- | --- | --- | --- | --- | --- | --- | --- | --- | --- | --- |
| **Measurement** | | **ROC curve analysis** | | **Predictive cut points** | | | | | | |
|  |  | **AUC (95% CI)** | ***P*** | **Cut point** | **Sensitivity** | **Specificity** | **PPV** | **NPV** | **Accuracy** | |
| 1 | First measure | 0.82 (0.79, 0.86) | <0.001 | 357 µmol/L (6.0 mg/dL) | 82.3% (76.3%, 87.3%) | 65.6%% (64.8%, 66.5%) | 3.8% (3.6%, 4.1%) | 99.6% (99.4%, 99.7%) | 65.9% (65.1%, 66.8%) | |
|  |  |  |  | 416 µmol/L (7.0 mg/dL) | 66.8% (59.9%, 73.2%) | 83.9% (83.2%, 84.5%) | 6.3% (5.7%, 6.9%) | 99.4% (99.2%, 99.5%) | 83.6% (82.9%, 84.2%) | |
|  |  |  |  | 476 µmol/L (8.0 mg/dL) | 44.7% (37.8%, 51.7%) | 94.1% (93.7%, 94.6%) | 11.0% (9.5%, 12.8%) | 99.1% (98.9 99.2%) | 93.4% (92.9%, 93.8%) | |
| 2 | Second measure | 0.83 (0.80, 0.87) | <0.001 | 357 µmol/L (6.0 mg/dL) | 86.3% (80.8%, 99.7%) | 53.0% (52.2%, 53.9%) | 2.9% (2.7%, 3.0 %) | 99.6% (99.4%, 99.7%) | 53.6% (52.7%, 54.4%) | |
|  |  |  |  | 416 µmol/L (7.0 mg/dL) | 76.0% (69.5%, 81.7%) | 75.4% (74.7%, 76.2%) | 4.7% (4.4%, 5.1%) | 99.5% (99.4%, 99.6%) | 75.4% (74.7%, 76.2%) | |
|  |  |  |  | 476 µmol/L (8.0 mg/dL) | 59.2% (52.2%, 66.0%) | 89.9% (89.3%, 90.4%) | 8.7% (7.7%, 9.7%) | 99.3% (99.1%, 99.4%) | 89.4% (88.8%, 89.9%) | |
| 3 | Average of both measures | 0.85 (0.82. 0.88) | <0.001 | 357 µmol/L (6.0 mg/dL) | 86.2% (80.6%, 90.7%) | 57.2% (56.3%, 58.1%) | 3.0% (2.9%, 3.2%) | 99.6% (99.5%, 99.7%) | 57.7% (56.8%, 58.5%) | |
|  |  |  |  | 416 µmol/L (7.0 mg/dL) | 75.4% (68.8%, 91.2%) | 79.9% (79.2%, 80.6%) | 5.6% (5.1%, 6.0%) | 99.5% (99.4%, 99.6%) | 78.9% (79.2%, 80.5%) | |
|  |  |  |  | 476 µmol/L (8.0 mg/dL) | 65.7% (58.7%, 72.2%) | 87.6% (87.0%, 88.2%) | 7.7% (7.0%, 8.5%) | 99.4% (99.3%, 99.5%) | 87.2% (86.7%, 87.8%) | |
| 4 | Highest of both measures | 0.85 (0.82, 0.88) | <0.001 | 357 µmol/L (6.0 mg/dL) | 88.7% (86.6%, 92.7%) | 48.1% (47.2%, 48.9%) | 2.7% (2.5%, 2.8%) | 99.6% (99.5%, 99.7%) | 48.7% (47.8%, 49.6%) | |
|  |  |  |  | 416 µmol/L (7.0 mg/dL) | 80.4% (74.3%, 85.6%) | 71.6% (70.8%, 72.4%) | 4.4% (4.0%, 4.7%) | 99.6% (99.4%, 99.7%) | 71.7% (70.9%, 72.5%) | |
|  |  |  |  | 476 µmol/L (8.0 mg/dL) | 66.3% (59.4%, 72.8%) | 87.6% (87.0%, 88.2%) | 7.9% (7.2%, 8.8%) | 99.4% (99.3%, 99.5%) | 87.2% (86.7%, 87.8%) | |
| All models were adjusted for sex, age, and cohort. BMI and renal function did not significantly contribute to the models (P>0.10) and were excluded as covariates. ROC = receiver operator characteristic; AUC = area under the curve; CI = confidence interval; PPV = positive predictive value; NPV = negative predictive value. Accuracy = defined as the number of true positive plus true negatives divided by the total number of participants. | | | | | | | | | | |
