# Supplementary material for: Is repeat serum urate testing superior to a single test to predict incident gout over time?
Source: PLoS One. 2022 Feb 1;17(2):e0263175. doi: 10.1371/journal.pone.0263175 (PMC8806054; doi:10.1371/journal.pone.0263175)
Supplement: S8 Table — (DOCX) [file pone.0263175.s010.docx]

| **S8. Heterogeneity statistics for difference in AUCs between cohorts** | | | | | | | |
| --- | --- | --- | --- | --- | --- | --- | --- |
|  | **AUC (95% CI)** | | | **Heterogeneity** | | | **Model-based AUC from Table 1** |
|  | **ARIC** | **CARDIA** | **FHS** | **Chi^2^** | **I^2^** | **FDR adjusted P-Value** |  |
| **Model 1 (Earliest)** | 0.82 (0.79, 0.86) | 0.81 (0.73, 0.90) | 0.71 (0.62, 0.79) | 6.32 | 68% | 0.16 | 0.81 (0.78, 0.84) |
| **Model 2 (Most recent)** | 0.83 (0.79, 0.86) | 0.85 (0.77, 0.92) | 0.74 (0.65, 0.82) | 4.53 | 56% | 0.40 | 0.83 (0.80, 0.86) |
| **Model 3 (Average)** | 0.84 (0.81, 0.88) | 0.84 (0.76, 0.92) | 0.75 (0.67, 0.83) | 4.28 | 53% | 0.48 | 0.84 (0.81, 0.87) |
| **Model 4 (Highest)** | 0.85 (0.81, 0.88) | 0.83 (0.75, 0.91) | 0.74 (0.66, 0.83) | 4.96 | 60% | 0.32 | 0.84 (0.81, 0.87) |
